# Impaired mucosal IgA response to SARS-CoV-2 in patients with inborn errors of immunity

**DOI:** 10.3389/fimmu.2026.1696834

**Published:** 2026-03-04

**Authors:** Fanglei Zuo, Samaneh Delavari, Sima Shokri, Yating Wang, Farhad Abolnezhadian, Sara Iranparast, Fereshte Salami, Samin Sharafian, Zahra Chavoshzadeh, Nima Rezaei, Hassan Abolhassani

**Affiliations:** 1 Division of Immunology, Department of Medical Biochemistry and Biophysics, Karolinska Institutet, Stockholm, Sweden; 2 Research Center for Immunodeficiencies, Pediatrics Center of Excellence, Children’s Medical Center, Tehran University of Medical Sciences, Tehran, Iran; 3 Department of Pediatrics, School of Medicine, Hazrat-e Rasool General Hospital, Iran University of Medical Sciences, Tehran, Iran; 4 Department of Pediatrics, Abuzar Children’s Hospital, Ahvaz Jundishapur University of Medical Sciences, Ahvaz, Iran; 5 Department of Immunology, Faculty of Medical Sciences, Ahvaz Jundishapur University of Medical Sciences, Ahvaz, Iran; 6 Pediatric Infections Research Center, Mofid Children’s Hospital, Shahid Beheshti University of Medical Sciences, Tehran, Iran

**Keywords:** breakthrough infection, inborn errors of immunity, mucosal immunity, primary antibody deficiency, SARS-CoV-2

## Abstract

**Background:**

Patients with inborn errors of immunity (IEI) often exhibit impaired responses to vaccination and infection, yet their systemic and mucosal antibody dynamics against SARS-CoV-2 remain incompletely understood.

**Methods:**

We investigated humoral immunity in 93 IEI patients recruited during the early phase of the pandemic, including pediatric patients with confirmed infection (n=64) and adult patients who received complete inactivated COVID-19 vaccination (n=29). Patients were classified as having primary antibody deficiency (PAD), combined immunodeficiency, or innate immune defects.

**Results:**

Receptor-binding domain (RBD)-specific IgG levels were comparable between infected children and vaccinated adults; however, PAD patients exhibited the weakest systemic and mucosal humoral responses, with markedly reduced salivary IgA. To validate these findings, we conducted a two-year follow-up of 15 PAD patients compared with 15 healthy controls. Despite regular intravenous immunoglobulin (IVIg) therapy, PAD patients had a ~4-fold higher re-infection rate than controls, with persistently low IgA and IgM in mucosal secretions. IVIg normalized IgG in serum, saliva, and nasal fluids but failed to restore mucosal IgA, which strongly correlated with re-infection frequency. Moreover, IgG responses to the latest emerging variants at the time of study (XBB.1.5, JN.1) declined in both groups, while mucosal IgA was more durable in controls.

**Conclusion:**

These findings underscore the critical role of mucosal IgA in protection and highlight persistent vulnerability in PAD patients despite IgG replacement therapy.

## Introduction

The dynamics of systemic and mucosal humoral immune responses against respiratory viruses such as severe acute respiratory syndrome coronavirus 2 (SARS-CoV-2) are influenced by individual immune system characteristics and genetic susceptibility (1). COVID-19 vaccinations predominantly elicit strong systemic immunoglobulin G (IgG) responses; however, their effectiveness against latest variants like Omicron is diminished, and they fail to provide robust mucosal immunity (2). Mucosal immunity, primarily driven by secretory IgA (sIgA), plays a critical early role in preventing viral entry, reducing replication, and potentially limiting transmission (3). Notably, SARS-CoV-2 infection induces higher mucosal neutralizing immunoglobulins compared to vaccination alone, underlining the importance of developing vaccines that effectively stimulate mucosal IgA responses (4).

We previously showed that patients with inborn errors of immunity (IEI), have a 4-fold higher mortality rate compared with the general population, with a significant number of severe and critical cases among children and young adults (5).In individuals with IEI, especially those with predominantly antibody deficiency (PAD) such as selective IgA deficiency (SIgAD), mucosal immunity can be severely compromised. SIgAD is the most prevalent IEI and is associated with recurrent mucosal infections, dysregulated gut microbiota, and increased inflammatory markers due to impaired epithelial barrier function and immune regulation (6). Studies of IEI patients infected with SARS-CoV-2 have demonstrated prolonged viral shedding and chronic infections in B-cell–deficient or agammaglobulinemic individuals, emphasizing the indispensable role of humoral immunity and secreted immunoglobulins in viral clearance and disease control (7).

Despite its clinical importance, mucosal immunity in PAD patients remains largely understudied. Here, we assess the correlation of systemic and secretory antibody profiles, including sIgA and IgA, in mucosal fluids (saliva, nasal fluid, tears) of IEI patients, particularly those with PAD, during the early and later phases of the COVID-19 pandemic. We further evaluate re-infection/breakthrough infection (BTI) rates and the correlation between mucosal antibody responses and infection susceptibility. By including healthy, multi-national controls with varied exposure histories, we aim to establish the baseline mucosal responses in immunocompetent individuals against which PAD patients’ responses can be compared.

## Materials and methods

### Study design

Available patients with IEI were recruited from the Iranian registry (8, 9) during the COVID-19 pandemic (December 2021–October 2023). In the early phase of the study (December 2021), recognizing that anti-SARS-CoV-2 antibody production is influenced by vaccination history, infection severity, and patient characteristics such as age and underlying immune defects, we applied two inclusion criteria for IEI patients: Pediatric patients without a history of COVID-19 vaccination but with confirmed infection by antigen or PCR testing. Adult patients who had completed a full course of inactivated COVID-19 vaccination (all received two doses of Sinopharm BBIBP-CorV) and had no documented medical history of infection at the time of recruitment. According to the national protocol during the pandemic, all patients with triad of COVID-19 have been tested, and we excluded those adults from the first phase of this study. Validating cohorts of patients were followed for two years collected during the late phase of the pandemic (until October 2023). Fifteen international COVID-19 vaccinated health controls were recruited and followed similarly to compare mucosal humoral response after full course vaccination by two to four doses of mRNA vaccine (BNT162b2 or mRNA-1273), or three doses of inactivated vaccine (BBIBP-CorV or CoronoVac), or the combination of mRNA vaccine and inactivated vaccine (10). The study was approved by the Theran University of Medical Sciences and Stockholm regional ethics committee (IR.RICH.REC.1400.041 and Dnr 2021-03217, respectively). Written informed consent was obtained from all participants and/or their legal guardians for the publication of any potentially identifiable genetic, clinical and immunologic data included in this article.

IEI patients were treated based on the Middle East and North Africa guidelines for IEI (11). Moreover, all studied cases were classified into three groups: Combined immunodeficiency (CID): T-cell defects including cellular and humoral immunodeficiencies, as well as combined immunodeficiencies with syndromic features. Predominantly antibody deficiencies (PAD): B-cell defects. Innate immune defects: Congenital phagocyte defects, autoinflammatory disorders, intrinsic/innate immunity defects, and complement deficiencies (12). To avoid the impact of severity of SARS-CoV-2 infection on humoral immune response, patients with severe or critical conditions were excluded (per World Health Organization guidelines for COVID-19 severity (13)). Data collected included demographics, clinical and molecular diagnoses (where available), treatments, and survival status. Samples (plasma extracted from blood, saliva, nasal fluid, tears) from IEI patients or healthy controls were collected 3–20 months after infection/BTI or 18–90 months after their last vaccination. Standardized self-collection methods were used, with appropriate processing and storage at −20°C or −80°C. Infection was confirmed by antigen or qPCR testing.

### Genetic evaluation and molecular diagnosis

Genomic DNA was extracted from the whole blood of the patients who consented to genetic evaluation and whole-exome sequencing (WES) was performed using a pipeline described previously (14, 15). Candidate variants were evaluated by the Combined Annotation Dependent Depletion (CADD) algorithm and an individual gene cutoff, given by using the Mutation Significance Cutoff (MSC) (16). The pathogenicity of all disease-attributable gene variants was re-evaluated using the updated guideline for interpretation of molecular sequencing by the American College of Medical Genetics and Genomics (ACMG) criteria (13, 17), considering the allele frequency in the population, computational data, immunological data and clinical phenotyping. We first prioritized the analysis of genetic changes in known IEI genes (12) showing a complete inheritance pattern and fulfilling the ACMG criteria.

### Preparation of SARS-CoV-2 receptor-binding domain protein

The RBD sequence of G614 (B.1 lineage, spike D614G, aa 319–541, GenBank: MN908947) was expressed using a baculovirus-free transient production system. Proteins were purified via HisTrap excel columns (Cytiva) and size exclusion chromatography (Superdex 200pg, Cytiva) (18, 19). RBDs of Omicron XBB.1.5, BA.2.86, and JN.1 were obtained from Sino Biologicals.

### Detection of total and SARS-CoV-2–specific antibodies

High-binding enzyme-linked immunosorbent assay (ELISA) plates (Corning, #3690) were coated overnight at 4°C with G614 RBD (1 μg/ml). After blocking, saliva, tears, and nasal fluid were applied at optimized dilutions (1:20 for saliva and nasal fluid and 1:10 for tears), as used in our previous mucosal antibody study. Plasma samples, when included, were diluted 1:1000 following established protocols (10, 20, 21). Bound antibodies were detected using isotype-specific HRP-conjugated secondary antibodies: goat anti-human IgM (Jackson ImmunoResearch), IgG (Southern Biotech), IgA (including IgA1-specific), and anti-secretory component antibodies (Sino Biological), each used at 1:3000–1:5000 depending on the antibody class, consistent with prior publications (10, 20, 21). Antibody levels were expressed in arbitrary units (AU/ml), based on a standard curve generated from serially diluted positive serum pools (22, 23). Concentrations of IgG, monomeric IgA1, and secretory IgA1 anti-RBD antibodies were determined using monoclonal standards (20). Data was normalized to total antibody concentrations to account for secretion flow variability (10, 24). To assess total immunoglobulin levels, separate plates were coated with capture antibodies for IgM, IgG, IgA, and secretory IgA (Sino Biological; 1 µg/ml), and samples were measured using respective secondary antibodies. For mucosal samples (saliva, nasal fluid, tears), antibody values were normalized to total immunoglobulin concentrations (expressed as percentage of total isotype) to account for differing mucosal flow and sampling yields, consistent with established methodology (1). Samples were detected with above-mentioned secondary antibodies and quantified against standard curves. Total protein content of mucosal samples was quantified using the Micro-BCA Protein Assay (Thermo Scientific), and in selected analyses, antibody levels were normalized to protein concentration (reported as mg antibody per g total protein), as performed in previous mucosal antibody studies of SARS-CoV-2. The positivity threshold for each ELISA was defined as the mean plus 2 standard deviations of pre-pandemic negative controls, as done previously for mucosal IgA and serum IgG assays. Samples with values below this threshold were considered negative (10, 20, 21). Mucosal antibody cut-off thresholds were defined using datasets from our previously established healthy control cohorts vaccinated with either inactivated or mRNA COVID-19 vaccines (10). Because the inactivated vaccine cohort was smaller, we conducted a direct comparison between the two healthy control groups to assess potential differences in mucosal antibody levels; these data were compared separately. Healthy controls recruited from internationally and consisted of young adult donors to enable age-matched comparison with the younger subset of IEI participants. All control samples were processed using identical laboratory procedures to ensure comparability across cohorts.

### Statistical analysis

Mann–Whitney U test was used for group comparisons; Wilcoxon matched-pairs signed-rank test was applied for paired data. Correlations were assessed using Spearman’s rank test. To assess the relationship between mucosal Ig levels and reinfection or BTI frequency, we implemented Poisson regression and mixed-effects models, which are more suitable for count-based outcomes and skewed predictor distributions. All statistical analyses were conducted using GraphPad Prism (v10) and R (v3.4.1; R Foundation, Vienna, Austria). A p-value <0.05 was considered statistically significant.

## Results

**Figure 1:**
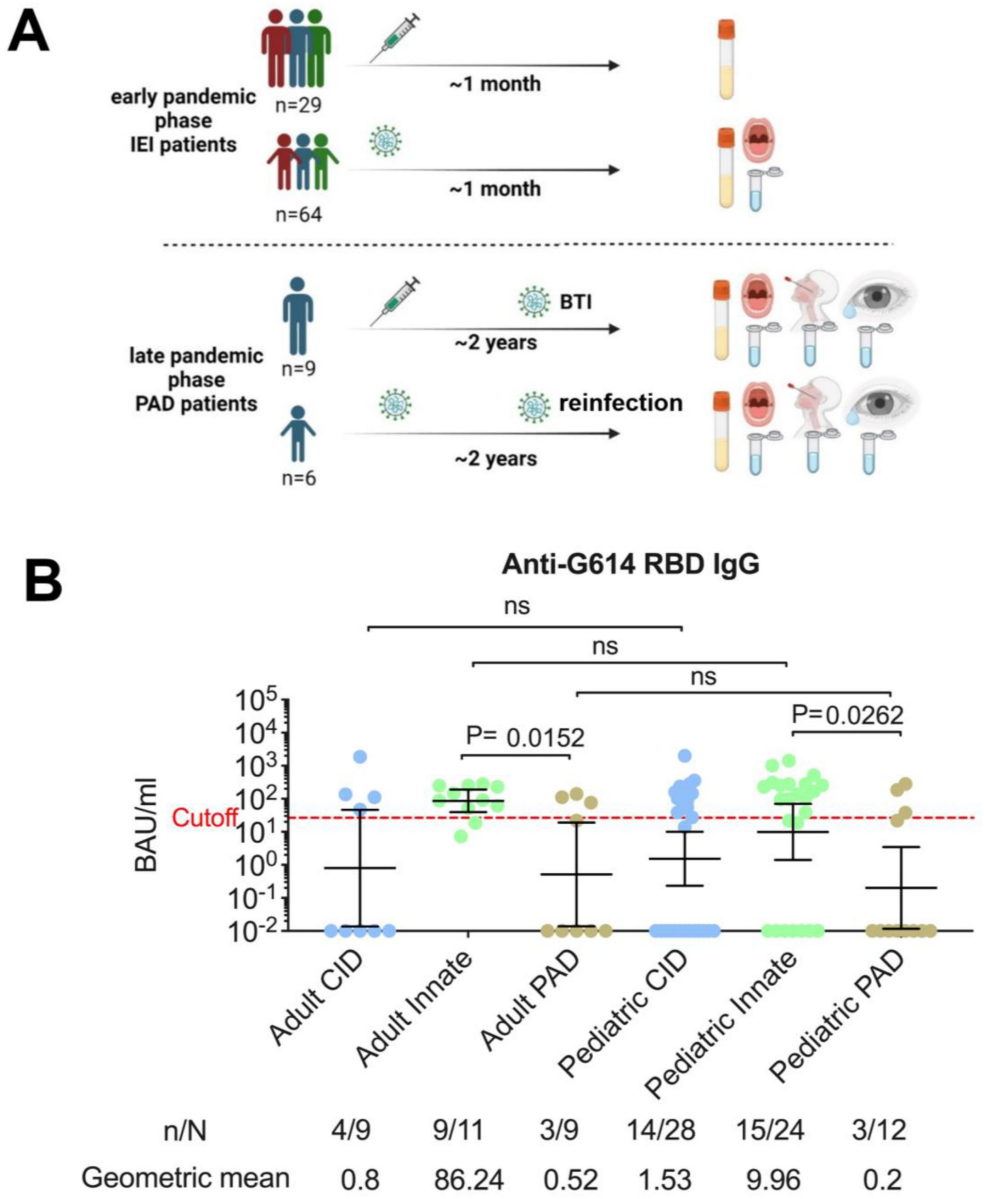
Schematic overview of the study design and quantification of G614 receptor-binding domain-specific IgG antibody levels in serum of IEI patients during the early phase of the pandemic. **(A)** The study assessed systemic and mucosal anti-SARS-CoV-2 responses in patients with inborn errors of immunity (IEI), including those with primary antibody deficiency (PAD), during both the early and late phases of the pandemic. **(B)** Serum IgG responses were measured in adult IEI patients with vaccination only and pediatric IEI patients with infection only. Each sample was run in duplicate, and each dot represents the mean value. Patients were classified into PAD, combined immunodeficiency (CID), or innate immune deficiency. Binding antibody levels are shown as geometric means with error bars representing 95% confidence intervals. Cutoff line has been generated based on the 2SD above the means of healthy controls sample collected prior to the pandemic (10, 20, 21). Statistical significance was evaluated using the two-sided Mann–Whitney U test for between-group comparisons. n = number of samples positive for immunoglobulin antibodies; N = total number of samples; BTI = breakthrough infection; ns = not significant.

A total of 93 available patients met the early phase inclusion criteria, comprising 64 pediatric IEI patients with non-severe SARS-CoV-2 infection (56.2% male; median age, 10 years; interquartile range [IQR] 8–15) and 29 adult IEI patients who had received COVID-19 vaccination (51.7% female; median age, 27 years; IQR 22–36) (**Figure 1A**). Patients were classified into three subgroups: combined immunodeficiency (CID, n=37), primary antibody deficiency (PAD, n=21), and innate immune defects (n=35) (**Supplementary Tables S1-S3**). The median interval between the last COVID-19 vaccine dose and sample collection in the adult IEI group was 21 days (IQR 18–37), while in the pediatric IEI group the median interval between SARS-CoV-2 infection and sample collection was 24 days (IQR 19–39). Among infected pediatric patients, most presented with mild to moderate symptoms during the acute phase, and only 7 (10.9%) required hospitalization.

Notably, the concentrations of plasma G614 RBD-specific IgG antibodies (targeting the B.1 lineage, spike D614G mutation) were comparable between vaccinated adults and infected pediatric patients (geometric mean 4.12 vs. 2.11 BAU/ml, p=0.88). However, stratification by immune defect revealed that PAD patients exhibited the lowest humoral responses, both in vaccinated adults (geometric mean 0.52 BAU/ml) and in infected children (geometric mean 0.20 BAU/ml, **Figure 1B**). Because mucosal IgA is crucial for protection following infection (3, 25), we further assessed salivary responses in a subset of SARS-CoV-2 infected IEI children (n=21). Consistent with systemic findings, PAD patients showed the weakest mucosal immunity, with G614 RBD-specific secretory IgA antibodies measured at 0.03 ng/ml (n=7, **Supplementary Figure S1**).

Based on these early observations, we conducted a two-year follow-up (through October 2023) of reinfection/BTI history in 15 PAD patients (validating cohort: 6 pediatric, 9 adults; independent of the first cohort, **Table 1**) and evaluated their systemic and extended mucosal anti-SARS-CoV-2 responses compared with 15 well-defined healthy controls (blood, saliva, nasal fluid, and tear samples) ^4^. None of the patients had active infection at the time of sampling. Despite regular intravenous immunoglobulin (IVIg) replacement therapy, PAD patients experienced a ~4-fold higher reinfection/BTI rate compared with healthy controls (35 episodes vs. 9 episodes; mean 2.3 ± 1.4 vs. 0.9 ± 0.4; p<0.001), with no significant difference between adult and pediatric IEI patients (2.6 ± 1.8 vs. 1.8 ± 0.7; p=0.37). The reinfection/BTI rate in IEI patients was also higher compared with the general Iranian inactivated-vaccine immunocompetent population (1.06 ± 0.03) evaluated during the same period and exposed to the same variants of concern^5^.

**Table 1:** Demographic characteristics of adult vaccinated and pediatric infected patients with primary antibody deficiency during the late phase of the pandemic.

ID	Sex	Group	Clinical diagnosis	Genetic diagnosis	Mutation	Adult/pediatric	WBC* (cell/μl)	Lym* (% of WBC)	CD19* (% Lym)	IgG* (mg/dl)	IgM* (mg/dl)	IgA* (mg/dl)	COVID-19 BTI/reinfection/	Days after the last symptoms	Vaccination	Days after the last vaccination
PL1	M	PAD	CVID	*CTLA4*	Het p.L28FfsX44	Adult	4930	35	4	300	68	6	7*####*	540	3d	570
PL2	M	PAD	CVID	*CTLA4*	Het p.G146R	Adult	5600	42	9	520	70	15	1*#*	870	3d	600
PL3	F	PAD	CVID	*CTLA4*	Het p.W165X	Adult	4820	33	5	324	65	8	3*#*	390	3d	570
PL4	F	PAD	CVID	Unsolved	–	Adult	6960	57	11	650	25	7	3*#*	300	3d	570
PL5	M	PAD	CVID	Unsolved	–	Adult	7800	50	8	245	17	5	3*##*	180	3d	720
PL6	M	PAD	CVID	Unsolved	–	Adult	7270	38	10	586	250	10	2*#*	210	3d	750
PL7	M	PAD	CVID	Unsolved	–	Adult	10600	50	10	137	23	26	1*#*	1050	4d	630
PL8	M	PAD	CVID	Unsolved	–	Adult	9520	34	11	336	10	3	2*##*	690	4d	690
PL9	F	PAD	CVID	Unsolved	–	Adult	8780	29	7.5	80	8	7	2*#*	870	2d	780
PL10	M	PAD	CVID	Unsolved	–	Pediatric	5800	39	5	420	0	0	2*#*	330	No	–
PL11	M	PAD	CVID	Unsolved	–	Pediatric	3200	75	17	448	20	21	1*#*	1000	No	–
PL12	M	PAD	CVID	*LRBA*	Hom p.R182X	Pediatric	6820	44	10	325	5	10	3*#*	380	No	–
PL13	F	PAD	CVID	*LRBA*	Hom p.S1605X	Pediatric	7720	17	5	200	15	5	2*#*	1180	No	–
PL14	F	PAD	CVID	Unsolved	–	Pediatric	4900	29	15	452	220	5	1*#*	270	No	–
PL15	F	PAD	CVID	Unsolved	–	Pediatric	8260	37	11	410	35	10	2*#*	30	No	–

PAD, predominantly antibody deficiency; CVID, Common variable immunodeficiency; d, doses of vaccine; WBC, White blood cells; Lym, Lymphocytes; CD19, B lymphocytes; Hom, homozygous; Het, heterozygous; BTI, breakthrough infection; M, male; F, female.

* Measured at diagnosis of CVID and before COVID-19 pandemic.

# Number of non-mild (moderate or severe) COVID infections required hospitalization.

**Figure 2:**
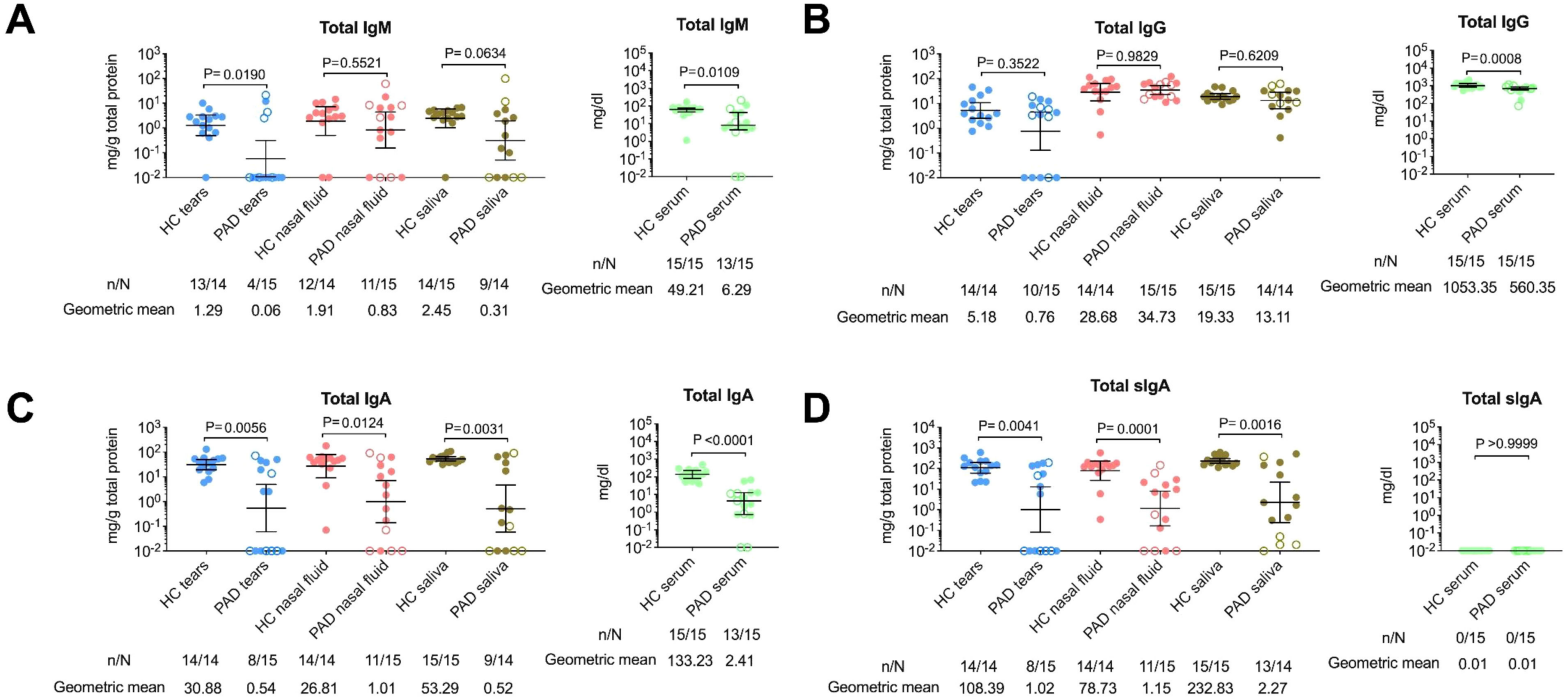
Total immunoglobulin levels in mucosal secretions of PAD patients compared with healthy controls during the late phase of the pandemic. **(A)** Total IgM, **(B)** total IgG, **(C)** total IgA, and **(D)** total secretory IgA (sIgA) were quantified in tears, nasal fluid, and saliva from patients with primary antibody deficiency (PAD) and healthy controls (HC). Each sample was run in duplicate, and each data point represents the mean value. Horizontal bars indicate geometric means with 95% confidence intervals (CIs). Filled dots represent vaccinated adult cases, and open dots represent infected pediatric PAD patients. Statistical significance was determined using the two-sided Mann–Whitney U test for between-group comparisons. n = number of samples positive for immunoglobulin antibodies; N = total number of samples.

**Figure 3:**
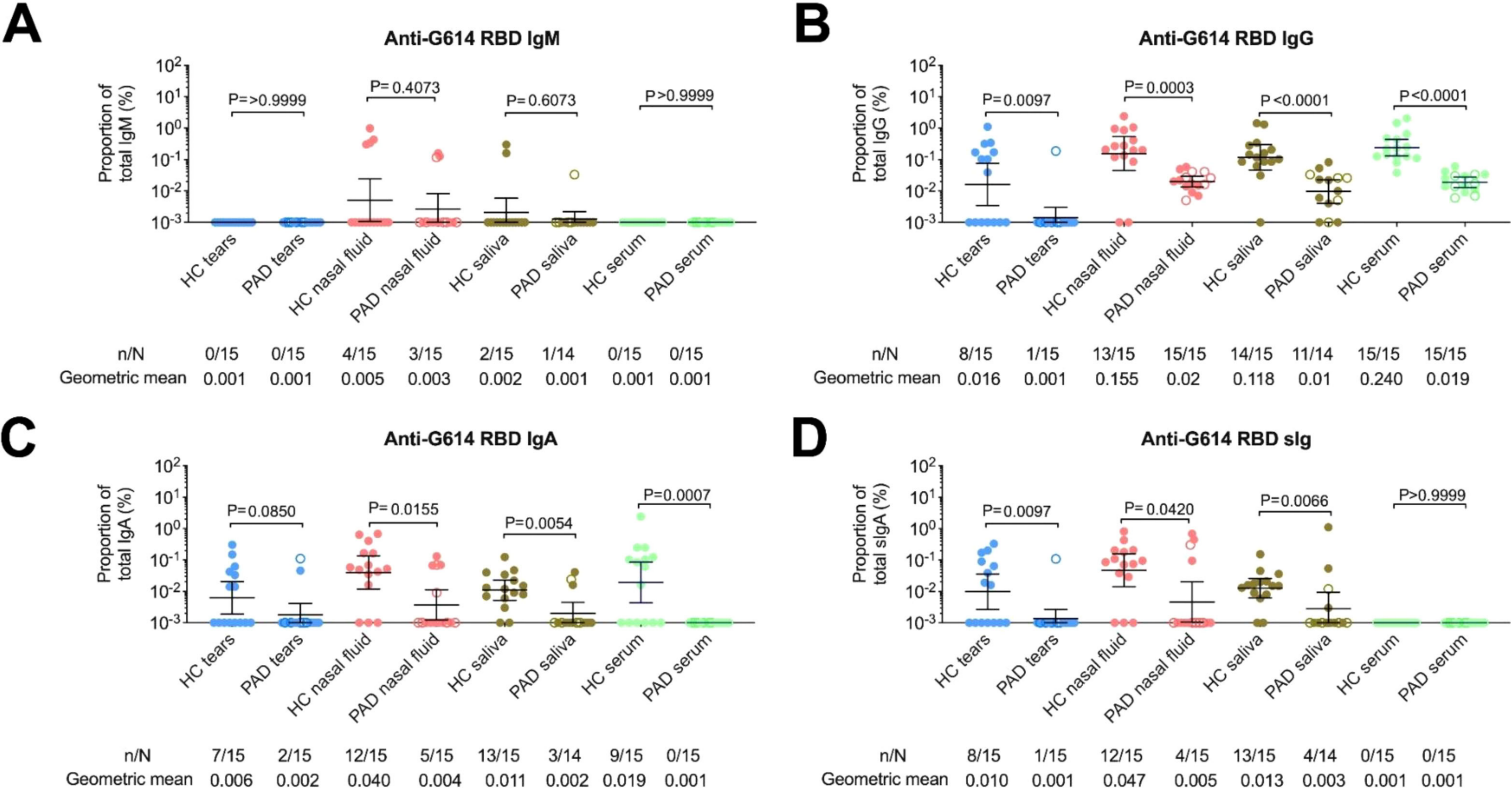
SARS-CoV-2 RBD-specific antibody responses in mucosal secretions and serum of PAD patients and healthy controls. **(A)** G614 RBD-specific IgM, **(B)** IgG, **(C)** IgA, and **(D)** secretory IgA (sIgA) were measured in matched saliva, nasal fluid, tears, and serum samples. To account for inter-individual differences in mucosal flow rates and sampling variability, RBD-specific antibody concentrations were normalized to the total immunoglobulin concentration of the corresponding antibody class in each sample. Horizontal bars indicate geometric mean with 95% confidence intervals (CIs). Filled dots represent vaccinated adult cases, and open dots represent infected pediatric PAD patients. Statistical significance was determined using the two-sided Mann–Whitney U test for between-group comparisons and the Wilcoxon matched-pairs signed-rank test for paired analyses. n = number of samples positive for the specified antibody; N = total number of samples. RBD = receptor-binding domain.

Serum analyses revealed IVIg therapy improved total IgG at the time of study compared with baseline at PAD diagnosis, but persistently low total IgA and total IgM (**Supplementary Figure S2A**). Moreover, IVIg therapy effectively normalized IgG levels in nasal fluid and saliva, with the levels observed in healthy controls and a partial effect in tears. However, IgA and IgM levels remained consistently lower in PAD patients than in healthy controls, except for normalized total IgM in nasal fluids (**Figure 2**). When focused more specifically on IgA as the main humoral response of mucosal layers, total IgA levels correlated strongly between serum and different mucosal secretions, as well as among mucosal sites (**Supplementary Figure S2B-D**). G614 RBD-specific IgA antibodies appeared to originate locally as secretory IgA, supported by direct correlations among their concentrations across mucosal sites (mainly in nasal fluid, r=0.92, p<0.001), consistent with prior findings in healthy individuals (10). Despite recurrent reinfection/BTI, PAD patients had significantly lower G614 RBD-specific mucosal IgA levels than healthy controls (p<0.05, **Figure 3**). Importantly, a strong negative correlation between mucosal IgA levels and reinfection/BTI frequency was observed exclusively in PAD patients, particularly in nasal secretions, independent of sampling time after the last infection (**Supplementary Figure S3**).

**Figure 4:**
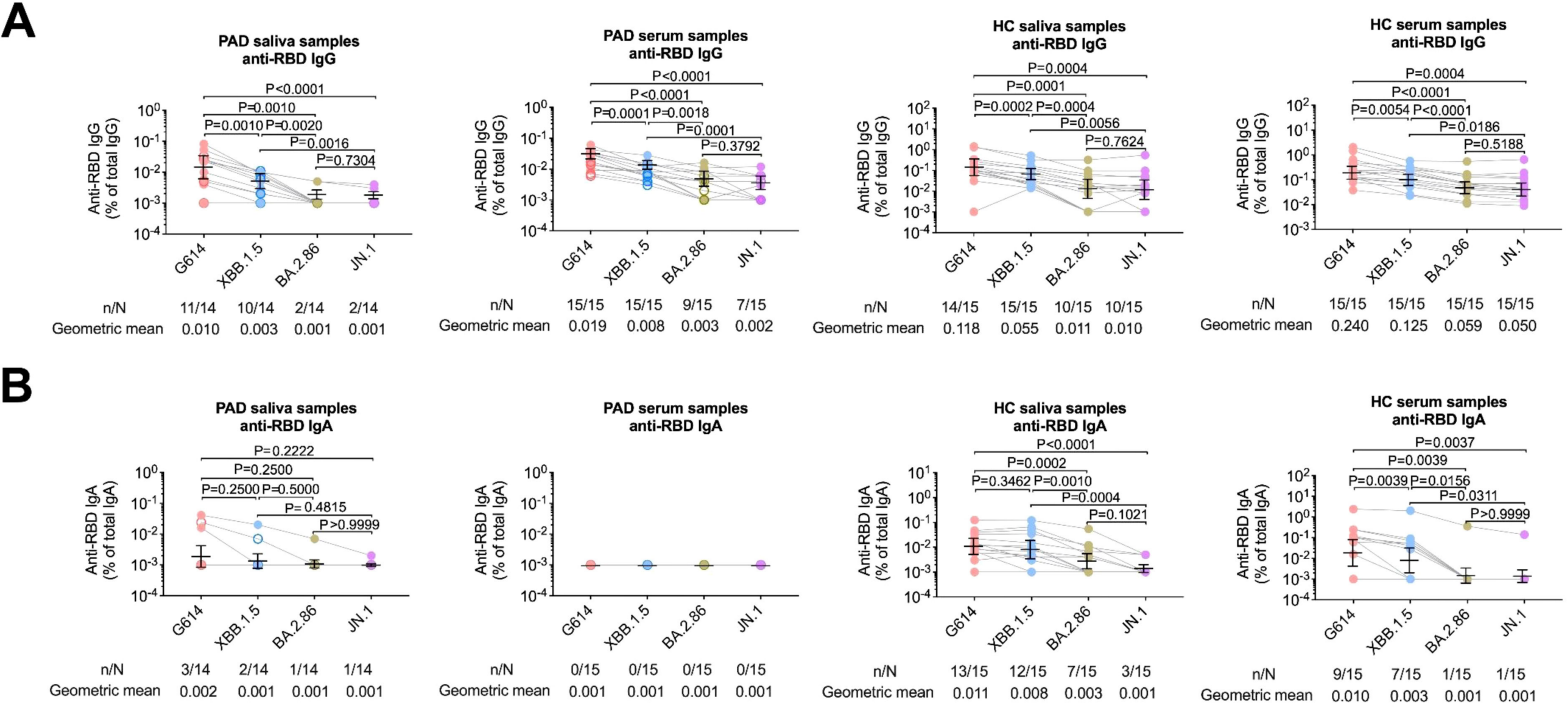
SARS-CoV-2 RBD-specific antibody responses in mucosal secretions and serum of PAD patients and healthy controls. **(A)** Comparison of RBD-specific IgG and **(B)** IgA antibodies targeting G614, XBB.1.5, BA.2.86, and JN.1 variants in matched saliva, nasal fluid, tears, and serum samples. All samples were assayed in duplicate, and each data point represents the mean value. Horizontal bars indicate geometric mean with 95% confidence intervals (CIs). Filled dots represent vaccinated adult cases, and open dots represent infected pediatric PAD patients. Statistical significance was determined using the two-sided Mann–Whitney U test for between-group comparisons and the Wilcoxon matched-pairs signed-rank test for paired analyses. n = number of samples positive for the specified antibody; N = total number of samples. RBD = receptor-binding domain.

We further examined the potential role of secretory IgM compensation^16^, since G614 RBD-specific IgM antibodies in mucosal fluids were not significantly different from healthy controls (**Figure 3A**). However, a positive correlation between G614 RBD-specific IgA and IgM suggested that the underlying immune defect in PAD patients impaired production of both antibody isotypes (**Supplementary Table S4**). Serum IgG specific to the RBD of XBB.1.5 and JN.1 (the dominant circulating variant at sampling) was lower and often near the limit of detection in both PAD patients and healthy controls compared with G614, with the steepest decline seen against JN.1. In mucosal samples, salivary RBD-specific IgA against JN.1 was significantly reduced, while responses to XBB.1.5 remained relatively stable in healthy controls (**Figure 4**). These findings align with our earlier results, showing stronger and more sustained mucosal IgA responses than systemic IgG responses (24). In contrast, PAD patients had low mucosal IgA levels, and in both groups, systemic IgG positivity was more common than IgA positivity. Additionally, although individuals in our inactivated vaccine group showed mucosal antibody levels comparable to those of mRNA vaccine recipients (**Supplementary Figure S4**), the small sample size and heterogeneity of controls mean these findings should be interpreted cautiously and not as proof of superior mucosal immunity from vaccination. Lastly, data from healthy individuals who received two doses of inactivated vaccine (24), demonstrated mucosal immune responses that were similar to or better than those caused by two doses of mRNA vaccine (**Supplementary Figure S4**). Therefore, these findings suggest that reduced responses in vaccinated PAD adults are mainly due to their underlying immune deficits rather than the vaccine platform.

## Discussion

Although PAD is the most common entity of IEI, the literature on residual antibody responses, particularly mucosal, in these patients remains limited. Our study contributes novel insights by evaluating both systemic and mucosal immune responses across multiple fluids (saliva, nasal fluid, tears), uncovering significant intra‐mucosal variability. For the first time, we documented the level of total and antigen-specific immune response and intra-mucosal variability in these patients as was suggested before based on the heterogeneity of B cell induction in different mucosal-associated lymphoid tissue (26). The total body pool of human IgA, consisting mainly as secretory IgA in mucosal areas, is higher than that for all other Ig isotypes and can be affected by all defects of early to terminal B cell differentiation. Secretory IgA is essential for preventing antigen entry, neutralizing pathogens, and maintaining mucosal homeostasis (25). Its early engagement at mucosal surfaces is a critical frontline defense. Over the past several years, few studies have examined the systemic humoral immune response to SARS-CoV-2 vaccination in patients with IEI, particularly those with PAD (27–33). These investigations consistently show that many IEI patients, most notably those with PAD, combined immunodeficiencies, and immune dysregulation defects in adaptive immune pathways, exhibit attenuated or delayed serologic responses, reduced vaccine-induced neutralizing activity, and variable T-cell immunity following mRNA or vector-based vaccination (34, 35). Prior work has also identified subgroups with markedly impaired vaccine responses, including patients with profound B-cell lymphopenia, class-switch defects, or altered germinal center dynamics, and has demonstrated that cellular immunity can remain partially preserved even when antibody production is minimal (27, 36). Despite these important contributions, nearly all published studies have focused exclusively on systemic immunity, with very limited assessment of mucosal antibody responses, especially secretory IgA after immunization with inactivated vaccine. Furthermore, the few available reports that evaluated SARS-CoV-2–specific antibodies in saliva or serum of IEI patients did not characterize antigen-specific mucosal IgA, nor did they compare responses across multiple mucosal sites. Thus, although the foundational literature has substantially advanced our understanding of systemic vaccine immunogenicity in IEI, a critical knowledge gap remains regarding mucosal immunity, which is essential for preventing viral entry and early transmission. Our study directly addresses this gap by providing a multi-compartment analysis of mucosal and systemic responses, enabling a more comprehensive interpretation of vaccine-induced immunity in PAD. Recent studies have highlighted the correlation between the level of salivary anti-RBD IgA antibodies and susceptibility to reinfection/BTI even in immunocompetent individuals (20), supporting the notion that PAD patients show a higher frequency of reinfection/BTI compared to other groups of IEI (50% vs. 16%) (37). Healy et al, also showed that PAD patients had the lowest SARS-CoV-2 spike-specific IgG responses in both saliva and serum compared to patients with other forms of IEI, however, they did not investigate the IgA level (38). Recent longitudinal studies demonstrate that high levels of mucosal IgA correlate with prolonged protection: for instance, individuals in the upper quartile of nasal IgA had about a 45% reduced risk of SARS-CoV-2 BTI (hazard ratio 0.55) over eight months after Omicron exposure, even as serum IgG waned (39, 40).

In contrast to robust systemic antibody responses, many vaccine platforms, including mRNA vaccines, elicit only transient and inconsistent mucosal IgA (41–43). One study showed that while most individuals had detectable salivary IgA two weeks post-vaccination, only around 30% retained it after the second dose, though secretory component–associated antibodies were more stable (41). These findings underscore the gap in mucosal protection provided by intramuscular vaccines. Natural infection profoundly boosts mucosal immunity (44). In recovered versus vaccinated-only individuals, nasal secretory IgA and neutralizing activity were much stronger, and in many cases were directly responsible for viral neutralization in the nasal mucosa (44, 45). Moreover, early and high salivary sIgA responses post-infection was associated with faster viral clearance and quicker symptom resolution, an effect comparable to having pre-existing vaccine-derived immunity (46). The importance of mucosal IgA is further highlighted by observations in individuals with SIgAD. Multiple cohort studies have shown that SIgAD increases the risk of severe COVID-19 several-fold, even up to 4-7× higher risk compared to individuals with normal IgA levels. Higher rates of infection and reinfection have also been documented in SIgAD populations (47).

Current data and previous investigations support a correlation between serum and mucosal IgA level in PAD patients (in contrast to healthy controls), even with the level at the time of diagnosis which can be used as indicator of cases at higher risk for BTI (48). This phenomenon is not necessarily due to lower diffusion of serum IgA to saliva but because the level of IgA produced is low in both compartments due to underlying immune defect. Our findings align with these insights and extend them to PAD patients, who similarly exhibit impaired mucosal IgA responses, higher reinfection/BTI rates, and extended vulnerability despite IVIg therapy. The fact that IVIg normalizes systemic IgG but does not fully compensate for mucosal IgA underscores a critical therapeutic gap. These patterns indicate that merely supplementing systemic immunity is insufficient. Alternative strategies, such as delivering IgA directly to mucosal surfaces, may be more effective (49). Experimental approaches, including nasally administered dimeric IgA (especially tailored to new variants) (24, 50), Omicron-convalescent plasma, or even breast milk–derived IgA, show potential (51). Correlation of mucosal immunity to other vaccines in the context of IEI particularly by measuring pneumococcal vaccine titers, which can indicate the functional humoral immunity in IEI should be consider in future studies. These data would have enabled to contextualize the mucosal immune defects observed relative to each patient’s ability to mount protective vaccine responses. Given that mucosal and systemic vaccine responses may diverge in immunodeficient states, future studies that integrate COVID-19 vaccine response, and other vaccine-specific titers with mucosal immunity profiling will be critical for determining whether the observed impairments are SARS-CoV-2–specific or indicative of a broader defect in immune responsiveness.

Limitations of this study include the relatively small PAD cohort in the late phase, which limits generalizability and may underestimate reinfection/BTI prevalence. The heterogeneous backgrounds of the healthy controls (10) may influence exposure dynamics and immune responses. The study also did not evaluate cellular immunity, which may be particularly relevant since T-cell responses can offer protection even when humoral immunity is weak. Future directions would be longitudinal, larger-scale studies evaluating both humoral and cellular immunity across mucosal and systemic compartments in PAD and other IEI subgroups. Clinical trials assessing mucosal vaccine candidates, especially nasal boosters, for their ability to induce protective sIgA responses and development of therapeutic interventions directly enhancing mucosal immunity (e.g., mucosal IgA delivery systems, IgA-enriched immunoglobulin products) should be conducted in future. Moreover, investigating the role of microbiota modulation (e.g., probiotics) to enhance mucosal IgA production is a challenging issue, since some evidence suggests such interventions may boost secretory IgA in other contexts (52). The recombinant RBD used in this study was produced using a baculovirus-free insect cell expression system, which can generate post-translational and glycosylation patterns distinct from those produced in mammalian cells. Because glycosylation can influence protein conformation and epitope accessibility, these differences may potentially affect antibody binding affinity. Although RBD proteins from Omicron XBB.1.5 and JN.1 was obtained from a commercial mammalian-based source, it is required to acknowledge that the non-mammalian expression of the G614 RBD represents is a limitation when directly comparing binding responses across variants in this study. While longitudinal sampling would have provided important insights into the durability of mucosal antibody responses and their relationship to the timing of prior infections, this study was not designed to include follow-up measurements. Our primary objective was to determine whether early mucosal humoral immunity could predict subsequent breakthrough infection, rather than to evaluate temporal dynamics. Future studies incorporating longitudinal designs will need to carefully control environmental exposure and standardize treatment and clinical care protocols, as these factors may substantially influence changes in mucosal immunity over time. Although we incorporated multiple healthy control datasets and performed additional comparisons between inactivated and mRNA vaccine recipients, the control group remains heterogeneous with respect to geographic origin, vaccine platform, and potential exposure to different SARS-CoV-2 variants. These factors may influence mucosal antibody levels and therefore limit direct comparisons with IEI patients. Future studies should establish harmonized, geographically matched control cohorts and include standardized vaccination and exposure histories. Such designs will allow more rigorous evaluation of mucosal immunity in IEI and support the development of more personalized vaccine strategies for this vulnerable population.

In conclusion, our findings suggest an association between higher mucosal IgA levels and reduced risk of SARS-CoV-2 infection in PAD patients, although we cannot infer causality in the absence of neutralization data. Our results indicate that intramuscular vaccination and IVIg may provide incomplete protection for these individuals, underscoring the potential value of strategies that better support mucosal immune responses. Further studies with larger cohorts and functional assays will be essential to define the precise contribution of mucosal IgA to protection in immunodeficient populations.

## Data Availability

The original contributions presented in the study are included in the article/**Supplementary Material**. Further inquiries can be directed to the corresponding author.
