# Supplementary material for: Impaired mucosal IgA response to SARS-CoV-2 in patients with inborn errors of immunity
Source: Front Immunol. 2026 Mar 4;17:1696834. doi: 10.3389/fimmu.2026.1696834 (PMC12996101; doi:10.3389/fimmu.2026.1696834)
Supplement: Supplementary file 1 [file DataSheet1.pdf]

Supplementary files

Impaired mucosal IgA response to SARS-CoV-2 in patients with inborn errors of immunity

Figure S1. Quantification of G614 RBD-specific antibodies in saliva of pediatric IEI patients during the early phase of the pandemic. (A) Salivary IgA and (B) secretory immunoglobulin responses were measured in pediatric IEI patients with infection only. Each sample was run in duplicate, and each dot represents the mean value. Patients were categorized into combined immunodeficiency (CID), primary antibody deficiency (PAD), or innate immune deficiency. Cut off lines have been generated based on 2SD above healthy control samples collected prior pandemic. Binding antibody levels are shown as geometric means, with error bars representing 95% confidence intervals. n = number of samples positive for immunoglobulin antibodies; N = total number of samples; RBD = receptor-binding domain.

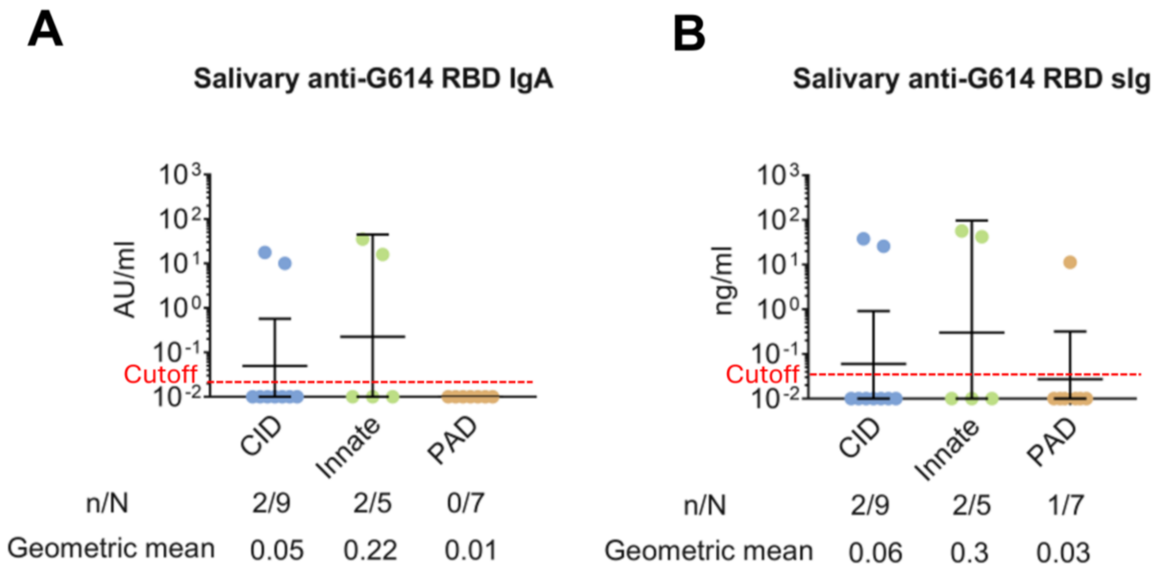

Figure S2. Correlation analyses of systemic and mucosal immunoglobulins in PAD patients during the late phase of the pandemic. (A) Comparison of immunoglobulin levels at the time of PAD diagnosis (pre-pandemic, red color boxplot) with late pandemic blood sample (blue color boxplot). (B) Correlation between total IgA and total secretory IgA (sIgA) in tears, nasal fluid, and saliva. (C) Correlation of total IgA across different mucosal fluids. (D) Correlation of total IgA between mucosal fluids and serum. Correlations were assessed using Spearman's rank test, with statistical significance defined as $p < 0.05$.

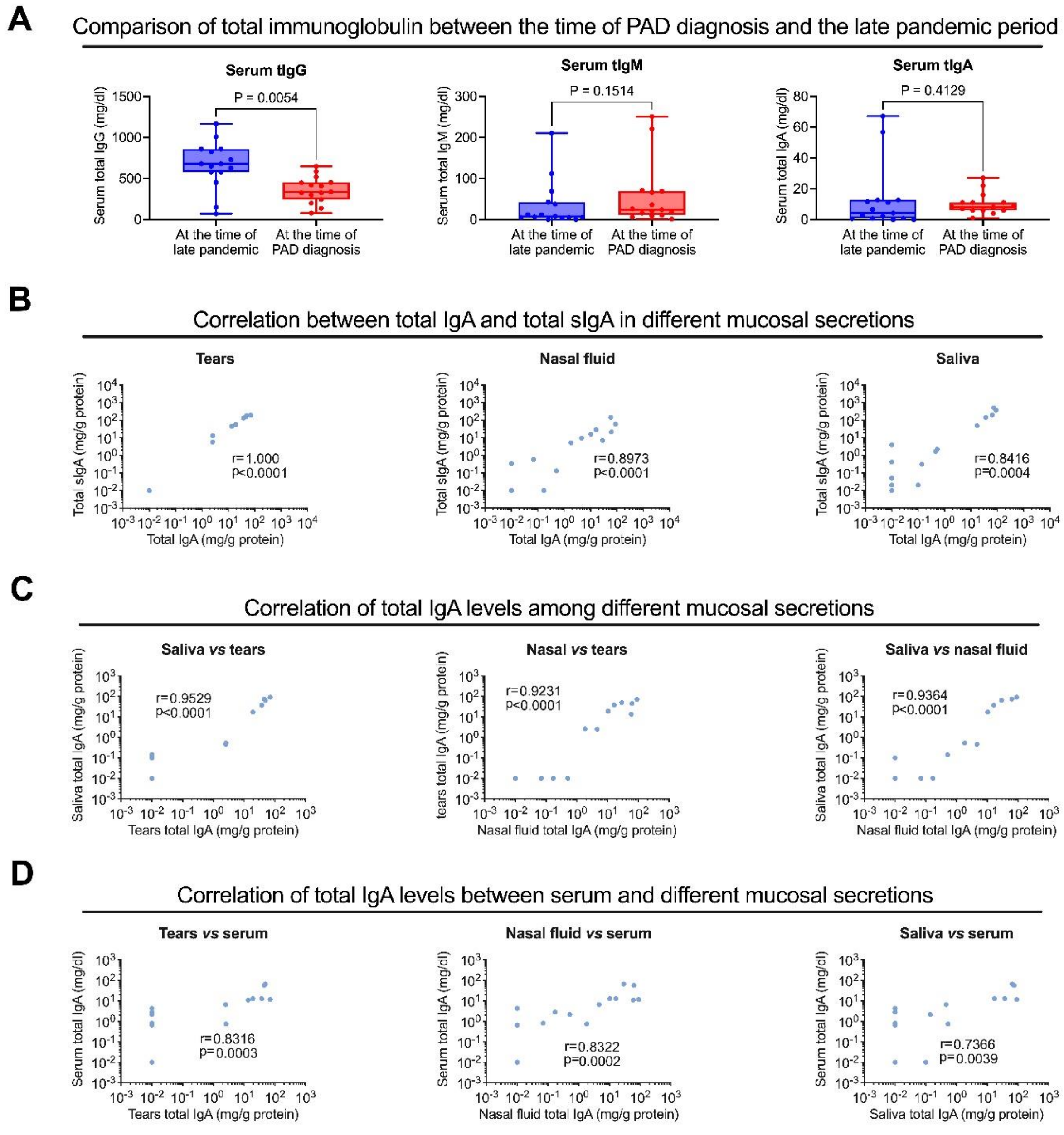

Figure S3. Correlation of mucosal SARS-CoV-2 RBD-specific IgA and secretory IgA with clinical outcomes. (A) Correlation between anti-G614 RBD-specific IgA/secretory IgA (sIgA) levels and breakthrough infection (BTI)/reinfection episodes. (B) Correlation between anti-G614 RBD-specific IgA/sIgA levels and sampling time in tears, nasal fluid, and saliva. Samples were run in duplicate. Correlations were assessed using Spearman’s rank test, with statistical significance defined as $p < 0.05$. HC = healthy controls; PAD = primary antibody deficiency.

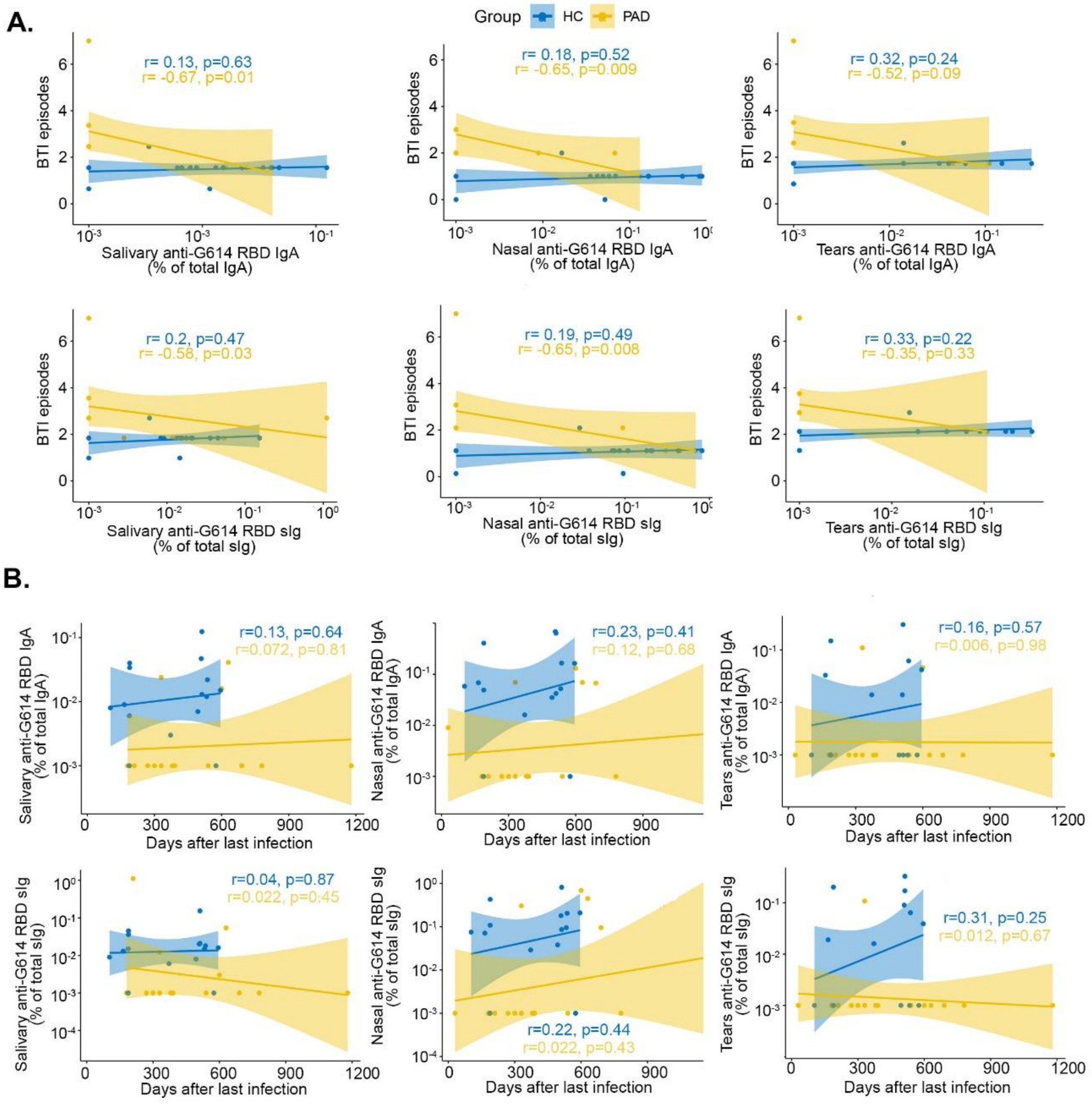

Figure S4. Anti-G614 RBD-specific antibody levels in plasma and saliva of vaccinated healthy individuals. (A) Plasma and (B) salivary antibody responses were measured in healthy individuals who received two doses of mRNA vaccine or two doses of inactivated vaccine. Cut off lines have been generated based on 2SD above healthy control samples collected prior pandemic. All samples were run in duplicate. Statistical significance was assessed using the two-sided Mann–Whitney U test for between-group comparisons, with significance defined as $p < 0.05$.

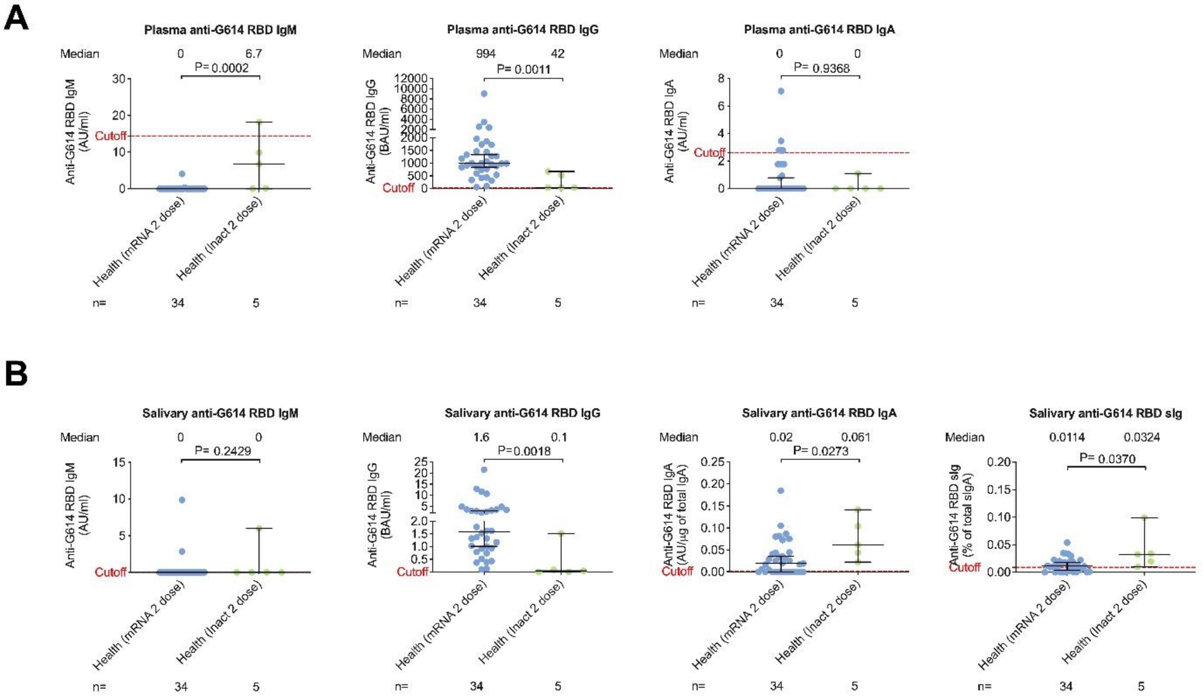

Table S1- Demographic characteristics of adult IEI patients vaccinated during the early phase of the pandemic.

ID	Sex	Group	Clinical diagnosis	Genetic Diagnosis	Mutation	Adult/ pediatric	Vaccine doses	Days after vaccination
PA01	M	CID	Syndromic CID	*ATM*	Hom p.L1238KfsX6	Adult	2d	37
PA02	F	CID	Syndromic CID	*BCL11B*	Het p.G578AfsX67	Adult	2d	21
PA03	F	CID	Syndromic CID	*ORAI1*	Hom p.P45AfsX44	Adult	2d	19
PA04	M	CID	Syndromic CID	*PMS2*	Hom p.D784N	Adult	2d	40
PA05	M	CID	Syndromic CID	*STAT3*	Het p.N466D	Adult	2d	10
PA06	M	CID	Syndromic CID	*POLA1*	Hem p.D303Y	Adult	2d	46
PA07	F	CID	Immune dysregulation	*RIPK1*	Het p.R37G	Adult	2d	20
PA08	M	CID	Immune dysregulation	*XIAP*	Hem p.R222X	Adult	2d	42
PA09	M	CID	Immune dysregulation	*FASLG*	Het p.V120F	Adult	2d	16
PA10	F	Innate	Autoinflammation	*MEFV*	Hom p.M680I	Adult	2d	18
PA11	M	Innate	Autoinflammation	*MEFV*	Hom p.M694V	Adult	2d	35
PA12	F	Innate	Autoinflammation	*MEFV*	Hom p.M694V	Adult	2d	18
PA13	M	Innate	Autoinflammation	*IFIH1*	Het p.R77L	Adult	2d	11
PA14	F	Innate	Chronic granulomatous disease	*NCF1*	Hom c.229+1del	Adult	2d	24
PA15	F	Innate	Chronic granulomatous disease	*NCF1*	Hom c.229+1del	Adult	2d	18
PA16	M	Innate	Complement deficiency	*CD55*	Hom p.A380LfsX13	Adult	2d	20
PA17	F	Innate	Complement deficiency	*SERPING1*	Het p.L243CfsX9	Adult	2d	53
PA18	F	Innate	Congenital neutropenia	*HAX1*	Hom p.W44X	Adult	2d	54
PA19	M	Innate	Congenital neutropenia	*HAX1*	Hom p,Q59X	Adult	2d	32
PA20	F	Innate	Mendelian susceptibility to mycobacterial disease	*IL12B*	Hom p.S176CfsX	Adult	2d	52
PA21	M	PAD	Agammaglobulinemia	*BTK*	Hem p.N72Ifs.X49	Adult	2d	19
PA22	F	PAD	Common variable immunodeficiency	*LRBA*	Hom p.D691H	Adult	2d	38
PA23	M	PAD	Common variable immunodeficiency	*LRBA*	Hom p. E59X	Adult	2d	21
PA24	F	PAD	Common variable immunodeficiency	Unsolved	-	Adult	2d	25
PA25	F	PAD	Common variable immunodeficiency	Unsolved	-	Adult	3d	16
PA26	M	PAD	Common variable immunodeficiency	Unsolved	-	Adult	2d	35

PA27	M	PAD	Common variable immunodeficiency	Unsolved	-	Adult	2d	14
PA28	F	PAD	Common variable immunodeficiency	Unsolved	-	Adult	2d	21
PA29	F	PAD	Common variable immunodeficiency	Unsolved	-	Adult	2d	22

PAD: predominantly antibody deficiency, CID: combined immunodeficiency, Hom: homozygous, Het: heterozygous, Hem: hemizygous, 2d: 2 doses of inactivated vaccines, M: male, F: female.

Table S2- Demographic characteristics of pediatric IEI patients with SARS-CoV-2 infection during the early phase of the pandemic.

ID	Sex	Group	Clinical diagnosis	Genetic Diagnosis	Mutation	Adult/ pediatric	COVID-19 infection	Days after onset of symptoms
PP1	F	CID	Non-syndromic CID	*RAG1*	Hom p.C358Y	Pediatric	Yes	12
PP2	F	CID	Non-syndromic CID	*RAG1*	Hom p.C358Y	Pediatric	Yes	18
PP3	M	CID	Non-syndromic CID	*CD40L*	Hem p.T29fsX36	Pediatric	Yes	18
PP4	F	CID	Syndromic CID	*ABCA1*	Hom p.K776N	Pediatric	Yes	15
PP5	F	CID	Syndromic CID	*ABCB4*	Hom p.S849X	Pediatric	Yes	18
PP6	F	CID	Syndromic CID	*ADA*	Hom p.D181N	Pediatric	Yes	20
PP7	M	CID	Syndromic CID	*ATM*	Hom p.Q2220X	Pediatric	Yes	13
PP8	F	CID	Syndromic CID	*ATM*	Hom p.R1875X	Pediatric	Yes	32
PP9	F	CID	Syndromic CID	*ATM*	Hom p.R23X	Pediatric	Yes	15
PP10	M	CID	Syndromic CID	*DNMT3B*	Hom p.D722E	Pediatric	Yes	39
PP11	M	CID	Syndromic CID	*DOCK11*	Hem p.I801V	Pediatric	Yes	54
PP12	M	CID	Syndromic CID	*DOCK8*	Hom c.2007+1G>C	Pediatric	Yes	22
PP13	M	CID	Syndromic CID	*FANCB*	Hem p.G666S	Pediatric	Yes	15
PP14	M	CID	Syndromic CID	*IKBKB*	Hom p.Q369R	Pediatric	Yes	18
PP15	M	CID	Syndromic CID	*IL2RG*	Hem p.S333RfsX1	Pediatric	Yes	14
PP16	F	CID	Syndromic CID	*KMT2D*	Het p.R755_P763dup	Pediatric	Yes	38
PP17	F	CID	Syndromic CID	*RBCK1*	Hom p.Q407R	Pediatric	Yes	15
PP18	M	CID	Syndromic CID	*SAMD9*	Het p.N720TfsX35	Pediatric	Yes	28
PP19	M	CID	Syndromic CID	*SAMD9*	Het p.S936X	Pediatric	Yes	38
PP20	F	CID	Syndromic CID	*STAT5B*	Het p.K582R	Pediatric	Yes	61
PP21	M	CID	Syndromic CID	*TGFBR2*	Het p.P129AfsX3	Pediatric	Yes	10
PP22	M	CID	Syndromic CID	*TGFBR2*	Het p.P129AfsX3	Pediatric	Yes	74
PP23	M	CID	Syndromic CID	*TTC37*	Hom p.P1270A	Pediatric	Yes	68
PP24	F	CID	Syndromic CID	*TTC7A*	Hom p.I380S	Pediatric	Yes	40
PP25	M	CID	Syndromic CID	*USP13*	Hom p.R480C	Pediatric	Yes	24
PP26	F	CID	Syndromic CID	*ZBTB24*	Hom p.R457X	Pediatric	Yes	14
PP27	M	CID	Immune dysregulation	*FOXP3*	Hem p.L428V	Pediatric	Yes	13

PP28	M	CID	Immune dysregulation	*MAGT1*	Hem p.W40CfsX5	Pediatric	Yes	48
PP29	F	Innate	Autoinflammation	*MVK*	Hom p.R388X	Pediatric	Yes	22
PP30	F	Innate	Autoinflammation	*NOD2*	Het p.L678WfsX85	Pediatric	Yes	31
PP31	M	Innate	Autoinflammation	*POLA1*	Hom p.L1454V	Pediatric	Yes	18
PP32	F	Innate	Autoinflammation	*SOCS1*	Het p.E152K	Pediatric	Yes	69
PP33	M	Innate	Autoinflammation	*IFIH1*	Het p.G965C	Pediatric	Yes	19
PP34	F	Innate	Complement deficiency	*C1QA*	Hom c.164-1G>T	Pediatric	Yes	29
PP35	M	Innate	Complement deficiency	*SERPING1*	Het p.G217VfsX	Pediatric	Yes	23
PP36	F	Innate	Chronic granulomatous disease	*CYBA*	Hom c.369+1G>A	Pediatric	Yes	20
PP37	M	Innate	Chronic granulomatous disease	*CYBB*	Hom c.252+5G>C	Pediatric	Yes	32
PP38	M	Innate	Chronic granulomatous disease	*CYBB*	Hem p.G364R	Pediatric	Yes	18
PP39	F	Innate	Congenital neutropenia	*ELANE*	Het p.G214R	Pediatric	Yes	35
PP40	F	Innate	Congenital neutropenia	*ELANE*	Het c.597+1G>A	Pediatric	Yes	22
PP41	F	Innate	Herpes simplex encephalitis	*TICAM1*	Het p.P367dup	Pediatric	Yes	75
PP42	F	Innate	Herpes simplex encephalitis	*TICAM1*	Het p.D233H	Pediatric	Yes	34
PP43	F	Innate	Leukocyte adhesion deficiency	*ITGB2*	Hom p.L8TfsX51	Pediatric	Yes	42
PP44	M	Innate	Mendelian susceptibility to mycobacterial disease	*IL12RB1*	Hom c.64+2T>G	Pediatric	Yes	9
PP45	F	Innate	Mendelian susceptibility to mycobacterial disease	*IL12RB1*	Hom c.64+2T>G	Pediatric	Yes	12
PP46	M	Innate	Mendelian susceptibility to mycobacterial disease	*IL12RB1*	Hom p.R173W	Pediatric	Yes	51
PP47	M	Innate	Mendelian susceptibility to mycobacterial disease	*JAK1*	Hom p.G307S	Pediatric	Yes	58
PP48	M	Innate	Mendelian susceptibility to mycobacterial disease	*JAK1*	Hom c.991-6T>C	Pediatric	Yes	67
PP49	M	Innate	Mendelian susceptibility to mycobacterial disease	*JAK1*	Hom c.991-6T>C	Pediatric	Yes	24
PP50	F	Innate	Predisposition to severe viral infection	*IFNAR1*	Hom p.H263fs14X	Pediatric	Yes	10
PP51	F	Innate	Predisposition to severe viral infection	*STAT1*	Het p.R274Q	Pediatric	Yes	44
PP52	M	Innate	Predisposition to severe viral infection	*STAT1*	Het p.R274Q	Pediatric	Yes	39
PP53	M	PAD	Agammaglobulinemia	*CD79A*	Hom p.P78RfsX103	Pediatric	Yes	19
PP54	M	PAD	Agammaglobulinemia	*IGHM*	Hom p.K64QfsX104	Pediatric	Yes	12
PP55	M	PAD	Agammaglobulinemia	*TOP2*	Het p.S738CfsX2	Pediatric	Yes	22
PP56	M	PAD	Hyper IgM syndrome	*AICDA*	Hom p.R92X	Pediatric	Yes	30
PP57	M	PAD	Hyper IgM syndrome	*AICDA*	Hom p.R92X	Pediatric	Yes	24
PP58	M	PAD	Common variable immunodeficiency	*CTLA4*	Het p.L28FfsX44	Pediatric	Yes	16

PP59	M	PAD	Common variable immunodeficiency	*PI3KR1*	Het c.836+1G>C	Pediatric	Yes	29
PP60	F	PAD	Common variable immunodeficiency	*PIK3CD*	Het p.E1045K	Pediatric	Yes	42
PP61	M	PAD	Common variable immunodeficiency	Unsolved	-	Pediatric	Yes	19
PP62	F	PAD	Common variable immunodeficiency	Unsolved	-	Pediatric	Yes	28
PP63	M	PAD	Common variable immunodeficiency	Unsolved	-	Pediatric	Yes	29
PP64	F	PAD	Common variable immunodeficiency	Unsolved	-	Pediatric	Yes	57

PAD: predominantly antibody deficiency, CID: combined immunodeficiency, Hom: homozygous, Het: heterozygous, Hem: hemizygous, M: male, F: female.

Table S3- Clinical and immunologic characteristics of IEI patients before SARS-CoV-2 infection or vaccination at the time of diagnosis of IEI.

ID	IEI groups	Major Clinical history	WBC (cells/µl)	Lymphocytes (cells/µl)	CD3+ T cells (cells/µl)	CD19+ B cells (cells/µl)	IgG (mg/dl)	IgM (mg/dl)
PP01	Pediatric	• Recurrent severe infections• failure to thrive• opportunistic infections• lymphopenia	2495	291	221	60	80	37
PP02	Pediatric	• Recurrent severe infections• failure to thrive• lymphopenia• opportunistic infections	2539	280	181	15	101	45
PP03	Pediatric	• Neutropenia• hyper-IgM phenotype• recurrent sinopulmonary infections• chronic diarrhea	5967	1488	945	327	277	1187
PP04	Pediatric	• Recurrent infections• hypogammaglobulinemia• orange tonsils• hepatosplenomegaly	5478	1816	1195	603	484	37
PP05	Pediatric	• Immune dysfunction• pruritus• recurrent infections• cholestasis	6522	1965	1437	725	916	97
PP06	Pediatric	• Recurrent infections• eczema• autoimmunity	4043	1205	650	310	453	68
PP07	Pediatric	• Ataxia• eczema• recurrent infections• lymphadenopathy	5995	1831	1219	645	751	112
PP08	Pediatric	• Ataxia •Telangiectasia • lymphadenopathy• recurrent infections• eczema• autoimmunity	6035	1781	1186	588	362	112
PP09	Pediatric	• Ataxia •Telangiectasia• lymphadenopathy• autoimmunity• eczema	5989	1848	1199	588	795	96
PP10	Pediatric	• LRI infection	4005	1805	1247	587	153	16
PP11	Pediatric	• Autoimmunity• eczema• skin infections	5973	1782	1248	633	772	85
PP12	Pediatric	• Eczema • Skin infections• autoimmunity• lymphadenopathy	6049	1806	1213	571	822	99
PP13	Pediatric	•Bone marrow failure •lymphadenopathy• recurrent infections• eczema	6099	1783	1198	552	837	166
PP14	Pediatric	• Eczema• recurrent infections• lymphadenopathy	6132	1849	1207	621	320	14
PP15	Pediatric	• Skin dermatitis• severe recurrent infections	2520	1430	830	615	57	13
PP16	Pediatric	• Recurrent LRI infections• autoimmunity• eczema	5960	1846	1158	603	837	114
PP17	Pediatric	• Severe eczema• autoimmunity• lymphadenopathy	6026	1825	1245	601	791	169
PP18	Pediatric	• Bone marrow failure• eczema• autoimmunity	5993	1835	1192	619	772	127
PP19	Pediatric	• Bone marrow failure• recurrent infections• lymphadenopathy• eczema	4042	1777	1238	553	848	168
PP20	Pediatric	• Recurrent respiratory and GI infections• eczema• autoimmunity	6023	1786	1191	627	753	106

PP21	Pediatric	• Recurrent sinusitis• autoimmunity• eczema	5974	1761	1181	645	781	115
PP22	Pediatric	• Autoimmune cytopenia• recurrent infections• eczema	10019	1751	1170	606	840	136
PP23	Pediatric	• Recurrent infections• autoimmunity• splenomegaly	6010	1814	1236	595	131	89
PP24	Pediatric	• Lymphadenopathy• autoimmunity• recurrent infections	4953	1761	1244	642	124	98
PP25	Pediatric	• Recurrent infections• eczema• autoimmunity	5960	1814	1203	606	807	160
PP26	Pediatric	• Recurrent URI infections	8967	1836	1210	603	352	15
PP27	Pediatric	• Recurrent infections• hepatomegaly• autoimmunity	9979	1760	1215	579	133	49
PP28	Pediatric	• Eczema• recurrent infections• autoimmunity	5958	1777	1216	647	250	52
PP29	Pediatric	• Severe dermatitis • recurrent LRI infections• lymphadenopathy• autoimmunity	15360	1837	1250	635	801	71
PP30	Pediatric	• Recurrent infections• autoimmunity• lymphadenopathy	6023	1786	NR	NR	NR	NR
PP31	Pediatric	• Eczema• recurrent infections• autoimmunity• lymphadenopathy	5977	1796	1183	558	843	128
PP32	Pediatric	• Recurrent respiratory infections• generalized lymphadenopathy• autoimmunity	6012	1805	1151	643	752	99
PP33	Pediatric	• Neutropenia • recurrent infections• lymphadenopathy• eczema	6005	1823	1175	641	778	163
PP34	Pediatric	• Recurrent URI• autoimmunity	5972	1754	NR	NR	NR	NR
PP35	Pediatric	• Skin allergic reaction• lymphadenopathy• recurrent infections	6045	1793	NR	NR	NR	NR
PP36	Pediatric	• Low NBT •deep infections • eczema• autoimmunity	9004	1777	1192	551	757	90
PP37	Pediatric	• Low NBT • recurrent infections• lymphadenopathy• autoimmunity	6043	1836	1247	617	756	89
PP38	Pediatric	• Low DHR • recurrent infections• lymphadenopathy• eczema	8042	1811	1201	559	832	136
PP39	Pediatric	• Neutropenia •recurrent infections• lymphadenopathy• autoimmunity	6014	1837	1209	629	807	167
PP40	Pediatric	• Neutropenia• lymphadenopathy• eczema• recurrent infections	5968	1816	1188	639	807	118
PP41	Pediatric	• Autoimmunity• recurrent infections• splenomegaly	6009	1841	1186	632	824	135
PP42	Pediatric	• Lymphadenopathy• eczema• autoimmunity	6038	1771	1207	633	830	141
PP43	Pediatric	• ITP• eczema• lymphadenopathy	5964	1772	1199	631	802	96
PP44	Pediatric	• MSMD• lymphadenopathy• eczema• recurrent infections	6018	1809	1166	561	765	144
PP45	Pediatric	• MSMD• recurrent infections• lymphadenopathy• eczema	5996	1824	NR	NR	NR	NR
PP46	Pediatric	• MSMD• eczema• lymphadenopathy• autoimmunity• recurrent infections	5951	1843	1207	607	799	108

PP47	Pediatric	• Autoimmunity• eczema• lymphadenopathy	5977	1799	1215	642	795	115
PP48	Pediatric	• Severe eczema• recurrent infections• lymphadenopathy	4045	1762	1226	573	823	149
PP49	Pediatric	• Lymphadenopathy •splenomegaly •autoimmunity	5966	1775	1247	583	837	102
PP50	Pediatric	• Severe viral infection •lymphadenopathy	7961	1764	1235	626	758	149
PP51	Pediatric	• JIA• lymphadenopathy• recurrent infections	5962	1834	1161	649	778	150
PP52	Pediatric	• Eczema• lymphadenopathy• autoimmunity	6010	1822	1230	557	785	84
PP53	Pediatric	• Recurrent infections• eczema	10019	1784	1179	25	11	19
PP54	Pediatric	• Recurrent respiratory infections• Polio vaccine infection	5976	1768	1190	35	5	0
PP55	Pediatric	• Recurrent otitis media• eczema• lymphadenopathy• autoimmunity	7975	1808	1244	640	22	9
PP56	Pediatric	• AIHA• IBD •recurrent infections• lymphadenopathy• eczema	6003	1770	1159	580	125	352
PP57	Pediatric	• Autoimmune hepatitis• lymphadenopathy• eczema	5972	1805	1198	635	59	392
PP58	Pediatric	• Enteropathy •eczema• recurrent infections• lymphadenopathy	6008	1833	1164	568	192	45
PP59	Pediatric	• Lymphadenopathy• recurrent infections• eczema	5962	1768	1205	587	115	133
PP60	Pediatric	• Recurrent LRI infections• autoimmunity• eczema	6035	1795	1163	614	96	16
PP61	Pediatric	• Lymphadenopathy• recurrent infections• autoimmunity	5953	1838	1158	604	292	38
PP62	Pediatric	• Autoimmunity• eczema• recurrent infections• lymphadenopathy and splenomegaly	5962	1821	1207	607	76	92
PP63	Pediatric	• Lymphadenopathy• recurrent infections• eczema	7019	1760	1178	622	182	13
PP64	Pediatric	• Eczema• recurrent infections• lymphadenopathy	5951	1803	1207	624	194	42
PA01	Adult	• Ataxia •Telangiectasia • autoimmunity• lymphadenopathy• eczema	6021	1809	1229	609	280	147
PA02	Adult	• Recurrent infections• autoimmunity• eczema	7017	1125	947	521	368	76
PA03	Adult	• Recurrent sinusitis• granulomatosis • eczema	5046	1790	1195	569	803	93
PA04	Adult	• Recurrent infections• lymphadenopathy• autoimmunity	8019	1817	1180	588	425	139
PA05	Adult	• Autoimmunity• recurrent infections• lymphadenopathy	6004	1834	1151	617	835	71
PA06	Adult	• Lymphadenopathy• autoimmunity• recurrent infections	5976	1792	1216	581	785	146
PA07	Adult	• GI infections• IBD• eczema	10960	1766	1193	626	761	79
PA08	Adult	• Lymphadenopathy• autoimmunity• recurrent infections	4990	1806	1175	619	258	33
PA09	Adult	• Recurrent URI and LRI infections• autoimmunity• lymphadenopathy	8024	1791	1174	563	797	92
PA10	Adult	• Splenomegaly• autoimmunity• recurrent infections	9523	1770	1234	617	806	165

PA11	Adult	• Lymphadenopathy• ITP• recurrent infections	10523	1760	1184	578	766	127
PA12	Adult	• Severe eczema• recurrent infections• autoimmunity	8452	1809	1248	619	750	73
PA13	Adult	• Neutropenia• recurrent infections• autoimmunity• lymphadenopathy• eczema	5983	1815	1173	590	802	77
PA14	Adult	• Low NBT• lymphadenopathy• recurrent infections• eczema	6988	1753	1185	565	834	94
PA15	Adult	• Low NBT • lymphadenopathy• recurrent infections• autoimmunity	5971	1850	1151	612	793	83
PA16	Adult	• Lymphadenopathy• eczema• recurrent infections	6034	1770	1247	615	833	147
PA17	Adult	• Recurrent diarrhea• autoimmunity• eczema	9048	1823	1175	560	832	106
PA18	Adult	• Neutropenia• eczema• autoimmunity• lymphadenopathy	6022	1818	1198	648	795	80
PA19	Adult	• Neutropenia • recurrent infections• autoimmunity• eczema• lymphadenopathy	7991	1831	1230	634	835	107
PA20	Adult	• MSMD • recurrent infections• eczema• lymphadenopathy	6042	1770	1228	554	845	104
PA21	Adult	• No tonsils • LRI• URI	5968	1815	1208	50	8	2
PA22	Adult	• Eczema• recurrent infections• Enteropathy	6036	1798	1232	625	342	26
PA23	Adult	• Enteropathy• eczema• recurrent infections	7005	1808	1157	561	259	17
PA24	Adult	• autoimmunity• eczema• recurrent infections	8016	1752	1206	617	287	45
PA25	Adult	• Allergic reaction• pneumonia • lymphadenopathy• autoimmunity	5961	1779	1163	624	23	17
PA26	Adult	• Recurrent infections• autoimmunity• eczema	4958	1775	1208	627	80	14
PA27	Adult	• Lymphadenopathy• eczema• recurrent infections	6022	1824	1238	649	157	17
PA28	Adult	• Pneumonia• autoimmunity• lymphadenopathy	5959	1760	1221	643	176	26
PA29	Adult	• Autoimmunity• chronic diarrhea• eczema• lymphadenopathy	8957	1804	1201	575	84	36

NR: Not reported in this specific patient. WBC; White blood cells, LRI: Lower respiratory infection, URI: upper respiratory infection, GI; gastrointestinal, MSMD: Mendelian Susceptibility to Mycobacterial Disease, NBT: Nitroblue Tetrazolium test. DHR: Dihydrorhodamine test, ITP: Immune thrombocytopenia, AIHA: Autoimmune hemolytic anemia, IBD: Inflammatory Bowel Disease, JIA: Juvenile Idiopathic Arthritis.

Table S4- Correlation between total and G614 RBD-specific IgA and IgM antibodies in serum, tears, nasal fluid, and saliva in 15 PAD patient vs 15 healthy controls. Correlations were assessed using Spearman's rank test, with statistical significance defined as $p < 0.05$.

Parameters	Groups	Serum	Saliva	Nasal	Tears
Total IgM vs. secretory IgA	PAD patients	NC	r=0.48, p=0.04*	r=0.30, p=0.28	r=0.28, p=0.32
	Healthy controls	NC	r=-0.43, p=0.11	r=0.91, p<0.001*	r=0.7, p=0.005*
Total IgM vs. total IgA	PAD patients	r=0.40, p=0.13	r=0.78, p=0.001*	r=0.23, p=0.39	r=0.53, p=0.19
	Healthy controls	r=0.29, p=0.25	r=-0.48, p=0.07	r=0.87, p<0.001*	r=0.29 p=0.30
Anti-G614 RBD IgM vs. secretory Ig	PAD patients	NC	r=-0.14, p=0.63	r=0.54, p=0.03*	NC
	Healthy controls	NC	r=0.13, p=0.65	r=0.29, p=0.29	NC
Anti-G614 RBD IgM vs. anti-G614 RBD IgA	PAD patients	NC	r=-0.14, p=0.62	r=0.46, p=0.05*	NC
	Healthy controls	NC	r=0.33, p=0.22	r=0.40, p=0.13	NC

PAD: predominantly antibody deficiency, NC: not calculatable.
